# Complex Full-Arch Treatment with Zygomatic Implants, Fully Digital Protocol with Scan Flag Intraoral Scanning, and 3D-Printed Temporary Reconstructions in a Periodontal Patient—A Case Report

**DOI:** 10.3390/biomedicines12112617

**Published:** 2024-11-15

**Authors:** Adam Nowicki, Karolina Osypko

**Affiliations:** 1 Diamante Clinica Dental Clinic, ul. Sportowa 48 A/C, 59-300 Lubin, Poland; adamusnowicki@gmail.com; 2 Dental Salon, Oral Surgery Academy, ul. E. Horbaczewskiego 53A, 54-130 Wrocław, Poland

**Keywords:** computer-aided implantology, digital smile design, face scanning, immediate loading, intraoral welding, surgical guide, zygomatic implants, 3D printing

## Abstract

**Background:** The following case report presents the treatment of a patient with severe maxillary atrophy and failing residual dentition. The patient has been diagnosed with stage IV grade C periodontitis, making this case challenging from the very beginning. **Methods:** The treatment plan was based on collecting and merging digital data: CBCT, a face scan, and an intraoral scan. Due to the advancement of the periodontal disease, the treatment was divided into three stages. The entire process was conducted in a digital manner, based on the concept of prosthetically driven implantology. Additionally, all prosthetic temporaries were planned via digital smile design. Stage I included extracting the residual dentition, placing four implants in the mandible, and the delivery of a 3D-printed upper removable denture. Stage II included placing two zygomatic implants, two anchored piriform rims, and one midline implant. Both arches were immediately loaded with the intraoral welding of abutments screwed to multiunit abutments and 3D-printed shells. Subsequently, in stage III, two milled ceramic superstructures combined with a titanium milled bar were delivered as a final screw-retained restoration with the application of scan flags (horizontal scan bodies) for intraoral scanning. **Results:** The aforementioned technologies can all be implemented and merged into one complex treatment plan combining high predictability, successful esthetics, and a reliable and accurate end result. Even though the concept of scan flags is relatively new, this case shows its potential and merit. **Conclusions:** This case represents the power of the digital approach as a helpful tool in the recreation of functional and esthetic smiles in compromised conditions in periodontal patients.

## 1. Introduction

Clinicians often face challenging situations in their professional work, and some of these situations are so complicated from the very beginning that the risk of failure is high. Analog and hand-driven surgery can be a huge success, though, for complex cases, our goal is to increase our success rate by careful, detailed planning and the precise application of digital tools. Intraoral scanning (IOS) technology itself has, of course, great potential; nevertheless, factors inherent to the oral cavity and the operator’s skills and understanding of the entire process can affect the quality of the image [1,2], and therefore, the end result of the treatment. The case presented below shows numerous applications of digital scanning (starting from diagnosis and planning to preparing the temporary and final reconstructions) as a suitable alternative to conventional impression making [2] and how it may be steadily implemented into everyday practice.

## 2. Patient Information and Clinical Findings

The reason the 53-year-old patient decided to visit a dental office was his complex dental situation that had been developing for years. He reported no systemic diseases and no regular medication.

**Figure 1:**
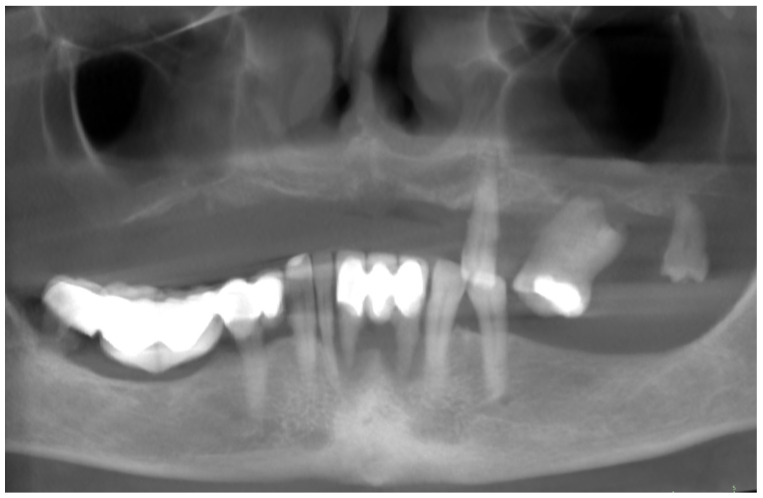
An orthopantomogram (OPG) view obtained using CBCT, showing the initial situation and signs of periodontitis (stage IV grade C). The residual dentition in the maxilla and advanced bone loss (almost 100% of the root’s length) around the incisors in the mandible are visible.

**Figure 2:**
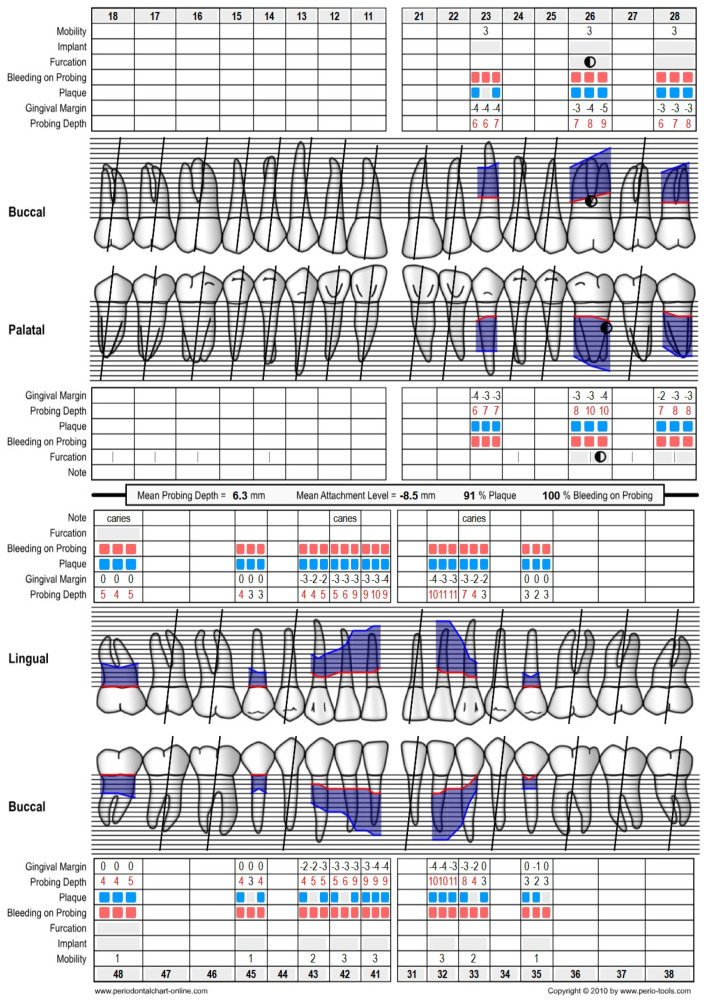
Periodontal chart of the initial situation, presenting the advancement of periodontitis and the unfavorable conditions of the remaining teeth. Blue and red rectangles mean the presence of plaque and bleeding on probing.

Intraoral examination and cone beam computed tomography (CBCT) revealed that the patient was using a removable partial denture in the upper arch and teeth-supported porcelain fused to a metal fixed bridge in the lower arch. The remaining and supporting teeth were failing due to abnormal mobility resulting from the advanced and rapid progress of periodontitis, classified as stage IV grade C [3], with more than 1.0% bone loss/age (Figure 1). The periodontal evaluation of the initial situation is summarized in the periodontal chart (Figure 2).

The patient requested some type of temporary prosthetic reconstruction for the healing period, which is common. He also informed us that, due to his social and professional activity, he would not accept being left visibly edentulous. After presenting the alternatives, he preferred to avoid sinus lifts or any other tissue grafting. Therefore, the final decision was to proceed with a treatment plan divided into three stages: Stage I involved a one-day All-on-X implant supported by a provisional prosthesis for the lower arch, along with a matching 3D-printed, mucosa-supported upper provisional prosthesis. Subsequently, after 3 months of the initial healing of the extraction sockets in the maxilla, a second surgery was conducted in stage II, with the implantation of two zygomatic, two piriform rims, and one midline implant, all immediately loaded with a temporary reconstruction created over a welded titanium structure. Next, after 6 months of the osseointegration period, this full mouth rehabilitation was completed with the final prosthetic work in stage III, conducted in a digital manner with the application of CAD/CAM technologies.

## 3. Therapeutic Intervention and Outcomes

### 3.1. Planning and Preparation for the Treatment

The treatment plan was made using digital data, including an intraoral scan, a face scan, and CBCT. Intraoral scans of the residual dentition were acquired by the application of a Dexis intraoral scanner (Dental Imaging Technologies Corp., Hatfield, PA, USA). The face scan was acquired by the application of Metismile (Shining 3D, Hangzhou, China). All of the data were merged with the assistance of Blue Sky Bio software (ver. 4.13; Blue Sky Bio, LLC, Libertyville, IL, USA).

**Figure 3:**
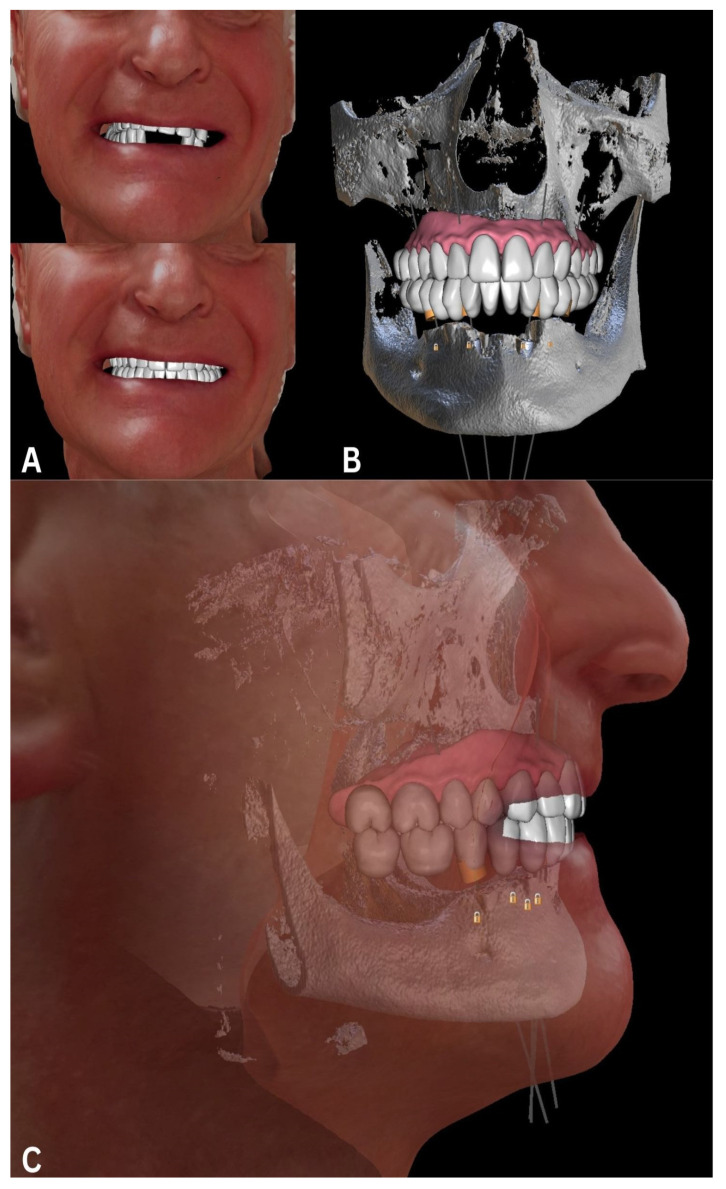
Planning and preparation with an ideal teeth arch superimposed over face scans (**A**) and CBCT (**B**), in accordance with Camper’s plane in side-face view (**C**). Data presented using Blue Sky Bio software (ver. 4.13; Blue Sky Bio, LLC, Libertyville, IL, USA).

The face scans enabled us to establish two new compatible arches, designed according to the patient’s esthetic characteristics and Camper’s plane (Figure 3).

**Figure 4:**
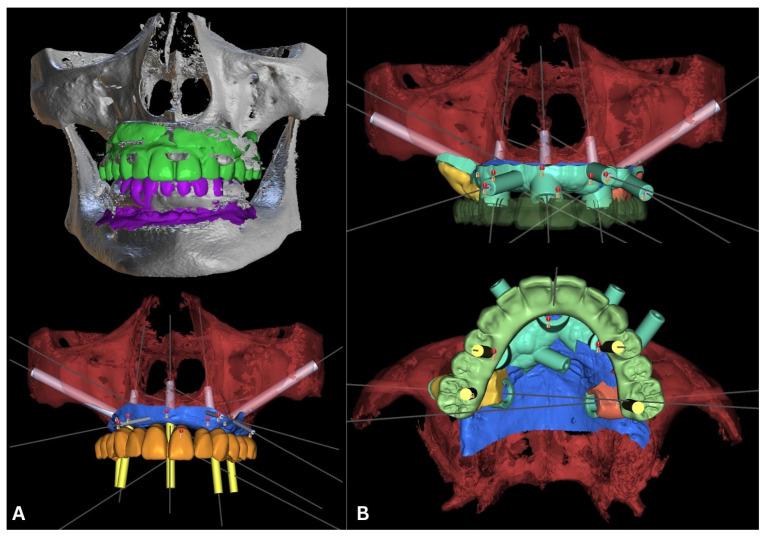
Planning for the maxilla. The bright green and purple .stls are scans of the initial situation (the remaining teeth and denture). The .stl of the maxilla (red) was acquired through segmentation in Blue Sky Bio. The blue is the mucosa, and the orange .stl is a new perfect teeth arch. The positions of all five implants (light gray cylinders) are visible together with the planned abutments (yellow cylinders) (**A**). The surgical guide is in turquoise, and the planned temporary reconstruction is in celadon, with visible localization of the abutment entrance points in yellow (**B**).

**Figure 5:**
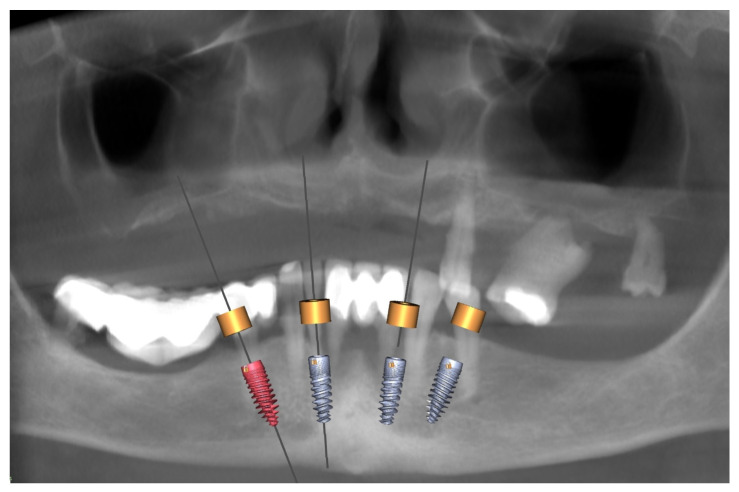
Planning for the mandible. An OPG image was obtained using CBCT. The planned positions of four implants were obtained using Blue Sky Bio software.

According to the rules of prosthetically driven implantology, implants were planned in the following regions: 16 (zygomatic), 14 (piriform), 11/21 (midline), 24 (piriform), 26 (zygomatic) (Figure 4), and 34 (tilted), 32, 42, and 44 (tilted) (FDI numbering system) (Figure 5).

A teeth-supported open surgical guide was designed with Forslab dental lab (Forslab, Warsaw, Poland) for the stage I surgery in the mandible for use with the fully navigated 2ingis system (2ingis, Brussels, Belgium). The removable upper denture, delivered on the same day after the extraction of the residual maxillary teeth, was designed using Meshmixer software (Meshmixer, Autodesk, Inc., ver:3.5.474, San Francisco, CA, USA).

All surgical guides, shells, and the upper removable denture, were 3D-printed inhouse with the use of a 3D printer and DLP technology (Phrozen mini 8k, Phrozen Tech. Co., Ltd., Hsinchu City, Taiwan) [4]. Light-curing resins SG and C&B N1 and NextDent Base Light Pink resins were supplied by Nextdent (NextDent B.V., Soesterberg, The Netherlands).

Also, for the stage II surgery, the initial mucosa-supported guide for pins and the following bone-supported guide for the placement of the implants were designed using the previously mentioned Blue Sky Plan and Meshmixer.

### 3.2. Surgery in the Mandible and Immediate Loading—Stage I

**Figure 6:**
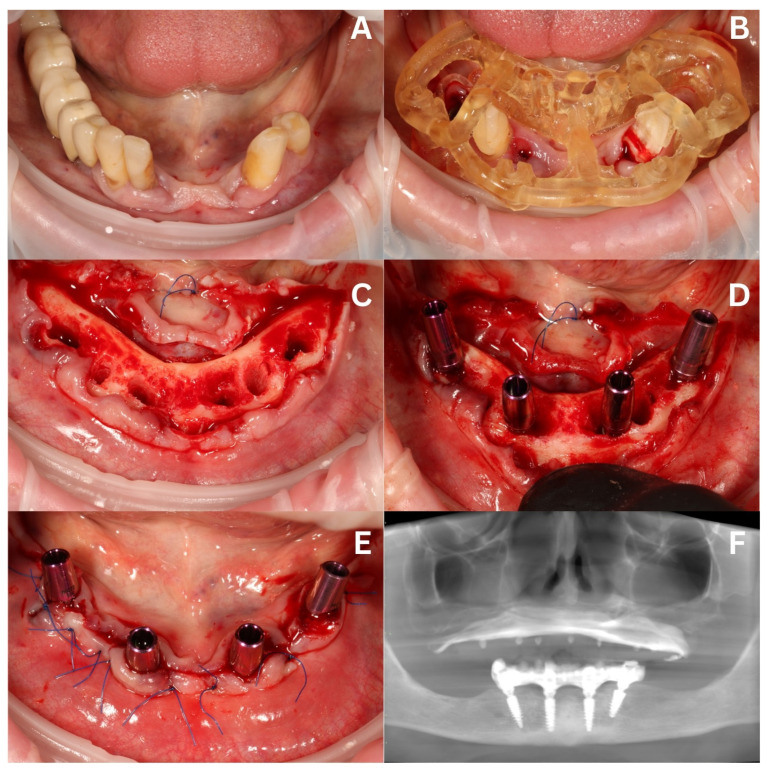
Stage I surgery—mandible. The initial situation (**A**). A 2ingis surgical guide placed on two canines to guarantee a static position for the osteotomies (**B**). The lingual flap retracted with a 5-0 nylon suture, alveolar ridge after bone reduction, osteotomies for implants, and extraction of canines (**C**). The situation after placing the implants, MUAs, and welding abutments (**D**) and after suturing the surgical field with 5-0 nylon mattress sutures (**E**). An OPG image obtained from post-op CBCT after intraoral welding and delivery of both temporaries (for the mandible and the maxilla) (**F**).

On the day of the stage I surgery, the patient was given local anesthesia: articaine + adrenaline (40 mg + 0.01 mg/mL). The remaining upper teeth were carefully extracted, and the sockets curetted and filled with A-PRFs. In the lower arch, the bridge and the remaining teeth were extracted, excluding the canines (temporal), as they were used as indexing and supporting pillars for the surgical guide (Figure 6A,B). After the initial osteotomy with ROOTT system burs (ROOTT, Trate Ag, Root, Switzerland) and a 2ingis open surgical guide, a full thickness flap was elevated with the lingual flap suspended by a nylon 5-0 suture (Resorba Medical GmbH, Nürnberg, Germany). The uneven bone ridge was flattened using the VarioSurg3 piezotome (NSK, Shinagawa City, Tokyo, Japan) (Figure 6C). Four ROOTT implants (ROOTT, Trate Ag, Root, Switzerland) were placed along with multiunit abutments (MUAs) and tall titanium abutments designed for welding (Figure 6D,E). The primary stability of the implants reached at least 45 Ncm. The next step was intraorally welding a 2.0 titanium wire to the welding abutments in order to create a rigid and passive framework. Afterward, the framework was inserted into a 3D-printed empty shell, resembling a teeth arch, and connected to it to obtain a long-term temporary prosthetic restoration. Next, a CBCT image was taken to confirm the final position of the mandible implants (Figure 6F).

**Figure 7:**
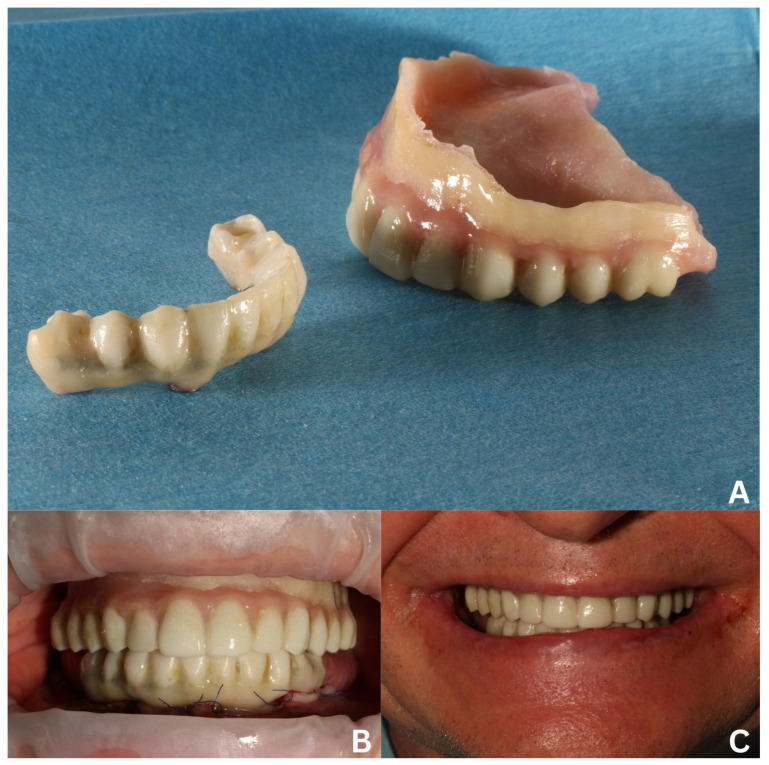
Temporary reconstructions delivered after the first surgery. Dismounted mandibular screw-retained temporary made on a welded titanium wire and teeth-resembling 3D-printed shell and maxillary temporary denture made in the image of an old, acrylic denture, relined with composite material for better adhesion (**A**). Both temporaries in intraoral (**B**) and extraoral views (**C**).

After dismounting the temporary, the mucosal side defects were filled with relining material to obtain an even and pontic shape, which promotes proper hygiene maintenance. With the reconstruction of the esthetics and functions for the period of osseointegration, the intramucosal side of the superstructure was polished [5], in contrast to the other surfaces covered with glaze (Optiglaze Clear, GC International AG, Luzern, Switzerland) (Figure 7).

A temporary 3D-printed denture for the maxilla was designed on the base of an old partial denture (which was scanned with Dexis). Next, it was lined on the mucosal side with composite material—Luxatemp (DMG Chemisch-Pharmazeutische Fabrik GmbH, Hamburg, Germany)—to fit the tissues better and to improve adhesion, and hence, proper functionality for the healing period (Figure 7A).

### 3.3. Surgery in the Maxilla and Immediate Loading—Stage II

**Figure 8:**
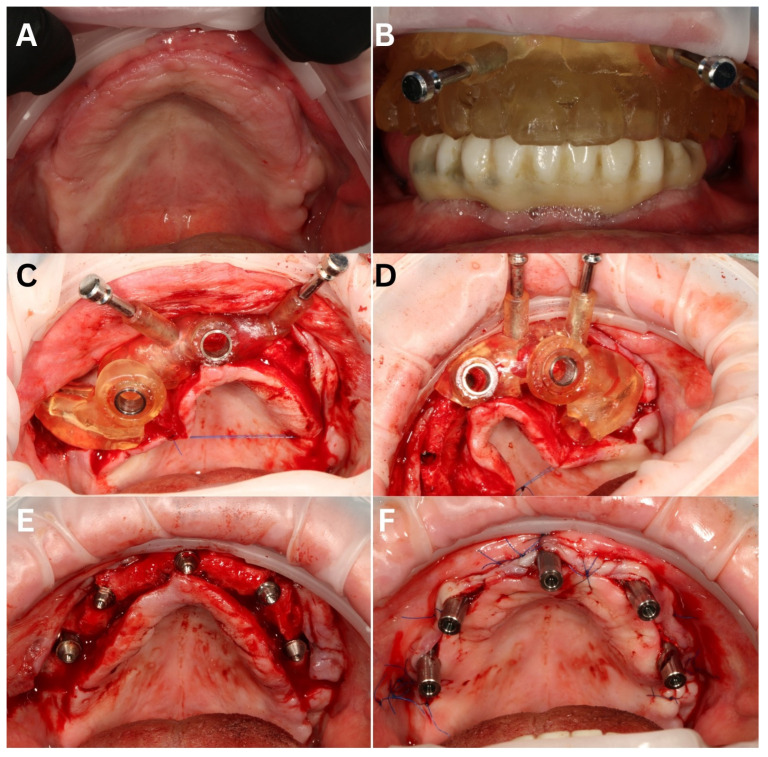
Stage II surgery—maxilla. The initial situation (**A**). The first surgical guide in the shape of a denture for drilling the pinholes (**B**), necessary to stabilize the implantological guide. Surgical guide divided into two parts: right (**C**) and left (**D**). On the right one, the lateral part is clearly visible, assisting navigation and guiding the osteotomy for the zygomatic implant. The situation after placing the implants and screwing in the MUAs (**E**) and with the welding abutments screwed on top of them (**F**).

Stage II of the surgical part took place after three months. Articaine + adrenaline (40 mg + 0.01 mg/mL) were used for infiltration and as a blocking anesthesia. Next, the primary surgical guide in the shape of a 3D-printed removable prosthesis (the one the patient had been using for the previous 3 months) was pinned to the bone to drill osteotomies for pinning the second, main surgical guide (Figure 8A,B). The opposing arch was used for indexing since there were no remaining teeth and the guide would have been supported fully by the mucosa, which is not precise enough for navigation purposes.

The full thickness flap was elevated and palatal ligation with a 5-0 nylon suture was applied (Resorba Medical GmbH, Nürnberg, Germany). The planning included five upper implants: two zygomatic, two anchored piriform rims, and one in the midline. The surgical guide was divided into two parts, and both of them had a guide hole for the midline implant (Figure 8C,D). Both parts of the guide were fully bone-supported and indexed with the use of pins. The piriform rim and the midline implant were placed with full navigation, while the zygomatic implants had an open part to prepare the lateral window to the maxillary sinus with a VarioSurg3 piezotome (NSK, Shinagawa City, Tokyo, Japan). 

After the lateral bone preparation, the Schneiderian membrane was lifted, and zygomatic drills were used to prepare the osteotomy in the zygomatic arch. The 3D-printed covering lid within the guide also assisted the navigation, since it was recreating the sleeve surface for the drill (Figure 8C).

**Figure 9:**
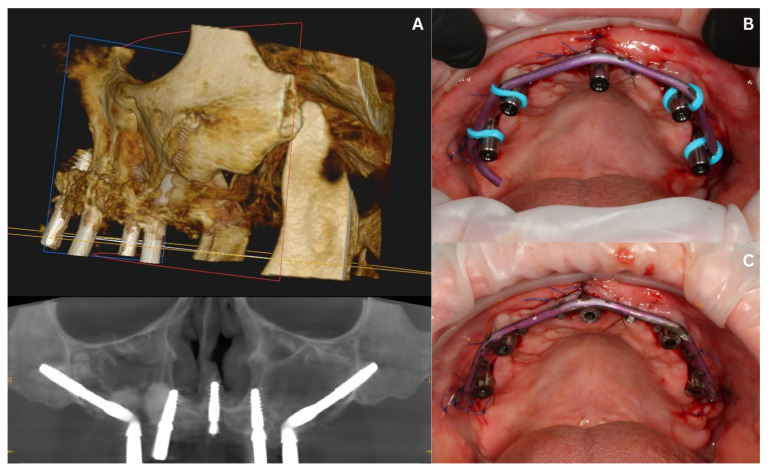
The situation after the second surgery: CBCT image showing the correct positions of the five implants in the maxilla (**A**); an intraoral view of the titanium wire prepared for welding (**B**); after welding and adjustments (**C**).

**Figure 10:**
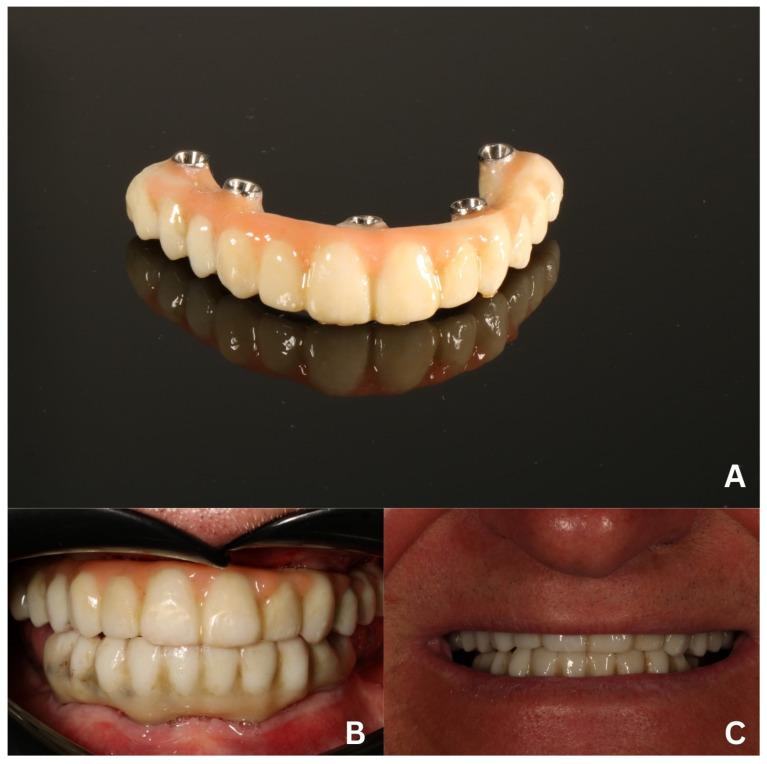
The temporary prosthetic reconstruction for the maxilla. Image after dismounting and finishing (coloring + polishing + glaze) (**A**), intraoral view (**B**) and extraoral view (**C**).

All the implants for the maxilla implantations were manufactured by ICX (ICX zygoma and ICX active, Medentis medical GmbH, Bad Neuenahr-Ahrweiler, Germany). The antrostomy was sealed with PRF membranes and covered with a mix of β-TCP and calcium sulphate—Ethoss (Ethoss Regeneration Ltd. Silsden, UK). Before closing up the operation field, the MUAs were screwed to the implant platforms (Figure 8E). Like in the lower arch, the MUAs were provided with welding abutments (Figure 8F and Figure 9A), to which the 2.0 titanium wire was welded intraorally—similarly to the mandible (Figure 9B,C). Afterward, the 3D-printed empty teeth arch-resembling shell was combined with a titanium framework with LuxaPick-up composite (DMG Chemisch-Pharmazeutische Fabrik GmbH, Hamburg, Germany) and Composite Primer by GC (GC International AG, Luzern, Switzerland), thus creating a rigid and durable long-term temporary restoration for the osseointegration period (Figure 10). 

CBCT images were also taken at this time to confirm the position of the implants (Figure 9A).

### 3.4. Final Reconstruction—Stage III

**Figure 11:**
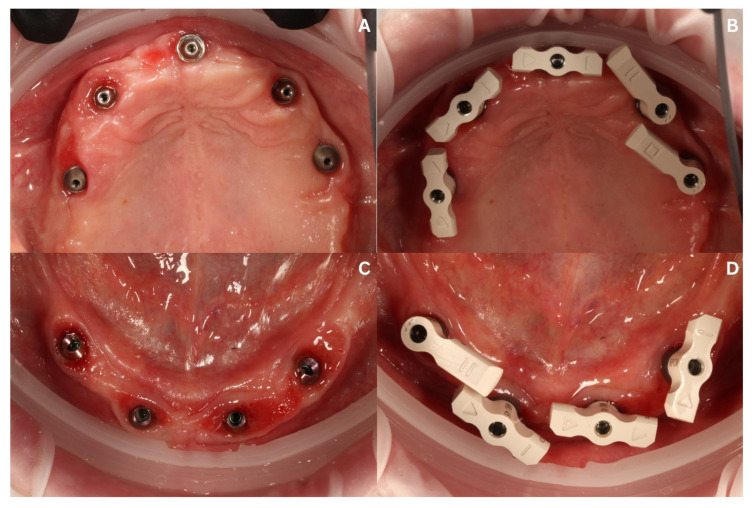
The clinical situation after 6 months of healing and during preparation for creating the final reconstruction via intraoral scanning (IOS). Intraoral view of ICX’s MUAs in maxilla (**A**), and with scan flags (SmartFlag by Apollo Implant Components, Pabianice, Poland) screwed in (**B**). Intraoral view of ROOTT’s MUAs in the mandible (**C**), and with scan flags (SmartFlags) screwed in (**D**).

After a healing period of 6 months after the maxilla implantations, a CBCT diagnosis was carried out (Carestream 8100 3D, Carestream Dental Germany GmbH, Stuttgart, Germany), and the temporary superstructures were dismounted to inspect the prosthetic field (Figure 11A,C).

Intraoral scanning was performed during the same appointment. Again, the Dexis scanner was used to capture the entire accessible prosthetic field and occlusion as a primary scan. Next, the reference scan for the mucosa and the emergence profiles of the MUAs were acquired along with the following scan body scan. For the proper acquisition of the position of the geometrical winged implants, horizontal scan flags (SmartFlag by Apollo Implant Components, Pabianice, Poland) (Figure 11B,D) were used due to their possibly enhanced scanning results in comparison to standard scan bodies, as they are similar to those presented by Zhang et al. [6]. 

**Figure 12:**
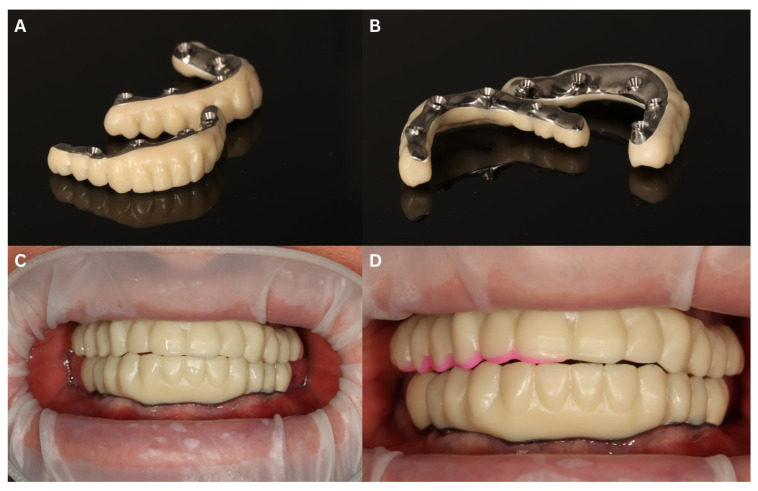
The first try-in of the final restoration. Milled titanium bar with 3D-printed shell superstructure (**A**,**B**) created for checking passive fit of the titanium bar and shape of the future teeth arch. Intraoral view after the try-in (**C**) and after minor changes in the lengths of the teeth, marked with pink flow composite material for better visibility and clear communication with a dental technician (**D**).

The dental laboratory provided a try-in framework and a 3D-printed try-in in the shape of a new restoration. After ensuring everything was passive, the lab provided a milled bar with a 3D-printed shell on top for further occlusal and passiveness testing (Figure 12A–C). At this point, the passiveness was checked via a Sheffield test. If the temporary reconstruction is properly fitted with MUAs and passive on the day of the surgery, its passive fitting on a 3D-printed model can be confirmed. Minimal incisal modifications were made and marked with a color flow-type composite in order to illustrate them for the technical laboratory (Figure 12D).

### 3.5. Results, Follow-Up, and Patient’s Perspective

**Figure 13:**
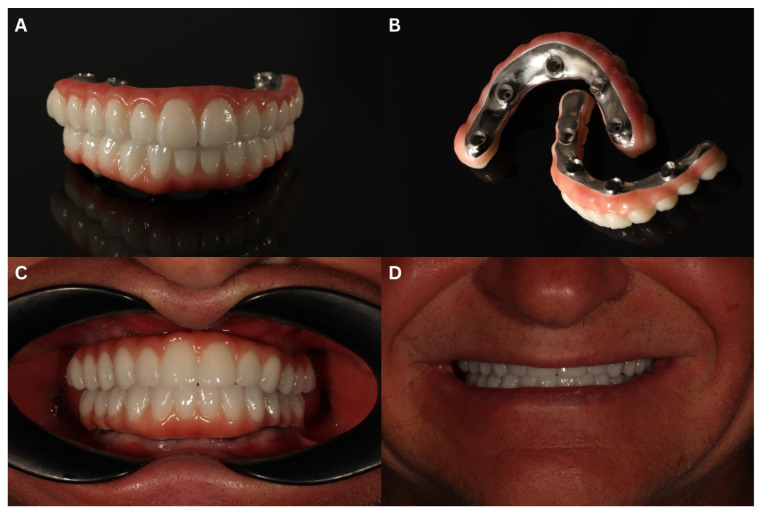
The final prosthetic reconstruction—a fully digitally made ceramic bridge placed on a milled titanium bar. Front view (**A**) and mucosa surface (**B**) before mounting. Intraoral (**C**) and extraoral (**D**) view after the delivery.

The last prosthetic stage was the delivery of a “taco-shaped” ceramic on top of a milled titanium bar and a screw attached to the MUAs (Figure 13A–C). Next, the static and dynamic occlusion was checked with the use of a digital OccluSense pressure meter (Baush, Germany GmbH, Hainspitz, Germany). At the last follow-up 7 months after the delivery of the final restoration, the result was esthetically and functionally satisfying to our patient (Figure 13D).

## 4. Discussion

Complex treatment plans containing full-arch/All-on-X implant cases with the immediate loading of temporary prosthetic reconstructions are considered challenging and advanced due to the need for making multiple medical decisions regarding biology, osseointegration, material engineering, occlusion, and esthetics [7]. All of the aforementioned need to be within the financial capabilities of the patient. 

### 4.1. Periimplantitis Prevention

The initial problem becomes even more difficult in the case of periodontal patients [8,9], who not only have compromised bone conditions due to bone and teeth loss but who are also more liable to periimplantitis and implant failure in the future [10,11]. This issue was addressed from the very beginning during the planning of the treatment, as even the implant connection and abutment type have significant influence on the end results and the predicted longevity of the implants. 

A study by Laleman and Lambert [12] regarding the risk of periimplantitis from an implant connection and abutment-type perspective suggests that a conical connection with platform switching and an abutment that is not frequently disconnected from the implant and has a prosthetic platform away from the bone are less prone to periimplantitis in the future.

These aspects, as a form of periimplantitis prevention, were taken into consideration while preparing the treatment plan.

Firstly, both implant systems used in our case (ROOTT and ICX) have a conical connection with platform switching. Secondly, the chosen abutment type (multiunit abutments) not only facilitated the passive fit of the prosthetic reconstruction but was also not meant to be disconnected from the implant ever again, as the only thing changing throughout the treatment time was the superstructure. According to a meta-analysis by Vatėnas and Linkevičius [13], frequent disconnection of an abutment significantly (and negatively) affects marginal bone loss. Moreover, MUAs move the prosthetic platform away from the bone level, thus lowering the risk of marginal bone loss [12]. Also, by creating more space for the soft tissue attachment and by maintaining its proper thickness and height, at least 3 mm [14] from the implant platform, MUAs help to maintain crestal bone stability and minimize the risk of implant loss [15,16].

### 4.2. Prosthetically-Driven Implantology

Full-arch cases have the advantage of the possible immediate loading of the implants, thus shortening the overall treatment time and providing the patient with a trial version of the future prosthetic reconstruction, which enables eating, smiling, social functioning, and helps to determine proper occlusion between the arches. 

In this particular case, the bone loss due to periodontitis resulted in poor conditions, atrophic maxilla, and limited options for placing the implants. One of the possible options was bone augmentation in the form of a sinus lift in the molar region of the maxilla and horizontal and/or vertical augmentation in the anterior maxilla and lateral mandible. An alternative solution was zygomatic, piriform, midline, and tilted implants. Both of the aforementioned options had their pros and cons, and both were possible to perform in accordance with medical standards; nevertheless, it was the patient who made the final decision. The treatment chosen and presented in this article, despite being less complicated to the patient, required even more advanced planning and the use of all surgical skills from the operator, due to the awareness of the possible complications [17], including, e.g., orbital cavity penetration [18].

The key to successfully completing such a case lies in detailed planning, dictated by DSD and Camper’s plane, and can be summarized as prosthetically driven implantology. While a few years ago, face scanners were considered a rarely used curiosity or an over-the-top gadget [19], it nowadays allows for planning whole treatments and utilizing the virtues of digital dentistry [7]. 

### 4.3. Digital Impressions for Final Prosthetic Reconstruction

As the surgical part of creating and printing guides and temporary restorations is widely known and implemented among clinicians, currently, the biggest challenge is to make accurate digital impressions for passive and well-fitted final prosthetic reconstruction on implants [20].

“Scan flags” or “horizontal scan bodies” are a novel approach to the matter of intraoral scan bodies (ISBs), designed especially for scanning full arches, where the lack of natural reference points (e.g., teeth), an abundance of unattached mucosa, and similar shapes of classic ISBs often result in difficulties during scanning and an inaccurate final scan [6], useless for a dental technician. Scan flags are a modified version of classic scan bodies that contain a longer, horizontal, flag-shaped part [6,21], which facilitates the continuity of scanning [22] and partially covers the mucosa, thus decreasing the risk of losing orientation by an intraoral scanner [23].

A recent systematic review by Pachiou et al. [24] suggests that extensional structures on an ISB, such as a flag-shaped processus, may improve the potential number of reference points and thus increase the accuracy of a digital impression. 

With improving technology and the constant development of new scanners and their software, more and more accurate scans will lead to better final prosthetic reconstructions. According to the systematic review by Abdelrehim et al. [25], a maximum vertical misfit of 160 μm or a horizontal misfit of 150 μm does not result in any mechanical complications. For biological complications, these values were 1 mm for vertical and 345 μm for horizontal misfit [25,26]. By implementing new tools, such as scan flags, it is possible to lower the risk of a misfit occurring and enable the repetitive manufacturing of full-arch reconstructions. Nevertheless, research and a scientific approach should underlie any improvement within the medical field. 

As pointed out by Pachiou et al. [24], there are many studies comparing different ISBs, their shapes, surfaces, height, inclination, and material, although it is difficult to objectively compare the results of these studies due to too many differences in the parameters, conditions, and different manufacturers of the ISBs. Pachiou even mentioned that sometimes different studies have drawn opposite conclusions. The SmartFlags used in this study have the advantage of being produced for many implant systems (such as ROOTT and ICX in our study); therefore, they may be a promising option for future comparative in vitro and in vivo studies, as one design of scan flags may be compared to the original, classic ISBs made by a given implant system manufacturer. Rational and objective results could then be obtained and potentially give clinicians some specific information about what is best for their patients.

The most prominent strength of this case is its complexity, while the two most significant limitations are the number of patients and the relatively short follow-up period. The latter was discussed among the authors until they reached the conclusion that early publication may provide more benefits for other researchers.

## 5. Conclusions

Nowadays, we have many useful new approaches and technologies to successfully handle even the most demanding clinical cases. In the age of digitalism, everything is expected to be completed quickly, precisely, and in a minimally invasive manner. Even though the operator’s skills and knowledge still determine the success rate of a whole mission, digital tools can help to push the boundaries even further. While the sole topics of full-arch treatments, zygomatic implants, remote anchorage, and immediate loading are becoming more and more common for dental professionals, their combination can lead to higher predictability and better outcomes with the application of digital tools. The authors believe that the future will bring improvements by replacing surgical guides with augmented reality (AR) and more innovations for the scanning procedure, perhaps by implementing stereophotogrammetry, although these areas still require more scientific analysis, comparison, and research. Nevertheless, in the end, the operator, their operating skills, and the preferred techniques play the main roles in the final outcomes, and no amount of technological support can substitute clinical experience.

## Data Availability

Data are contained within the article.

## Abbreviations

CAD-CAM	Computer-aided design–computer-aided manufacturing
CBCT	Cone beam computed tomography
IOS	Intraoral scan
ISB	Intraoral scan body
MUAs	Multiunit abutments
OPG	Othopantomogram

## References

[1] Pereira A.L.C., Medeiros V.R., da Fonte Porto Carreiro A. (2021). Influence of implant position on the accuracy of intraoral scanning in fully edentulous arches: A systematic review. J. Prosthet. Dent..

[2] Pereira A.L.C., Curinga M.R.S., Segundo H.V.M., da Fonte Porto Carreiro A. (2023). Factors that influence the accuracy of intraoral scanning of total edentulous arches rehabilitated with multiple implants: A systematic review. J. Prosthet. Dent..

[3] Tonetti M.S., Greenwell H., Kornman K.S. (2018). Staging and grading of periodontitis: Framework and proposal of a new classification and case definition. J. Periodontol..

[4] Nowicki A., Osypko K., Kurzawa A., Roszak M., Krawiec K., Pyka D. (2024). Mechanical and Material Analysis of 3D-Printed Temporary Materials for Implant Reconstructions-A Pilot Study. Biomedicines.

[5] Mazurek-Popczyk J., Nowicki A., Arkusz K., Pałka Ł., Zimoch-Korzycka A., Baldy-Chudzik K. (2022). Evaluation of biofilm formation on acrylic resins used to fabricate dental temporary restorations with the use of 3D printing technology. BMC Oral Health.

[6] Zhang T., Yang B., Ge R., Zhang C., Zhang H., Wang Y. (2024). Effect of a Novel ‘Scan Body’ on the In Vitro Scanning Accuracy of Full-Arch Implant Impressions. Int. Dent. J..

[7] Nowicki A., Osypko K. (2024). Digital Smile Design, Vast Bone Reduction with Surgical Guide and Intraoral Welding—A Case Report. Dent. Res. Oral Health.

[8] Hamasni F.M., El Hajj F., Abdallah R. (2018). Single Sitting Surgical Treatment of Generalized Aggressive Periodontitis Using GTR Technique and Immediate Implant Placement with 10-Year Follow-Up. Case Rep. Dent..

[9] Nowicki A., Osypko K. (2023). Digital Workflow in Full Mouth Rehabilitation with Immediate Loading, Intraoral Welding and 3D-Printed Reconstructions in a Periodontal Patient: A Case Report. Reports.

[10] Rokaya D., Srimaneepong V., Wisitrasameewon W., Humagain M., Thunyakitpisal P. (2020). Peri-implantitis Update: Risk Indicators, Diagnosis, and Treatment. Eur. J. Dent..

[11] Heitz-Mayfield L.J.A., Heitz F., Lang N.P. (2020). Implant Disease Risk Assessment IDRA-a tool for preventing peri-implant disease. Clin. Oral Implant. Res..

[12] Laleman I., Lambert F. (2023). Implant connection and abutment selection as a predisposing and/or precipitating factor for peri-implant diseases: A review. Clin. Implant Dent. Relat. Res..

[13] Vatėnas I., Linkevičius T. (2021). One abutment one time vs. repeatable abutment disconnections in implants, restored with cemented/screw retained fixed partial dentures: Marginal bone level changes. A systematic review and meta-analysis. Stomatologija.

[14] Tomasi C., Tessarolo F., Caola I., Wennström J., Nollo G., Berglundh T. (2014). Morphogenesis of peri-implant mucosa revisited: An experimental study in humans. Clin. Oral Implant. Res..

[15] Tavelli L., Barootchi S., Avila-Ortiz G., Urban I.A., Giannobile W.V., Wang H.-L. (2021). Peri-implant soft tissue phenotype modification and its impact on peri-implant health: A systematic review and network meta-analysis. J. Periodontol..

[16] Linkevicius T., Apse P., Grybauskas S., Puisys A. (2010). Influence of thin mucosal tissues on crestal bone stability around implants with platform switching: A 1-year pilot study. J. Oral Maxillofac. Surg..

[17] Kämmerer P.W., Fan S., Aparicio C., Bedrossian E., Davó R., Morton D., Raghoebar G.M., Zarrine S., Al-Nawas B. (2023). Evaluation of surgical techniques in survival rate and complications of zygomatic implants for the rehabilitation of the atrophic edentulous maxilla: A systematic review. Int. J. Implant Dent..

[18] Al-Nawas B., Aghaloo T., Aparicio C., Bedrossian E., Brecht L., Brennand M., Chow J., Davó R., Fan S., Jung R. (2023). ITI consensus report on zygomatic implants: Indications, evaluation of surgical techniques and long-term treatment outcomes. Int. J. Implant Dent..

[19] Yang S., Wu L., Alabkaa B., Lepidi L., Yue L., Li J. (2024). Intraoral scanner-based virtual facebow transferring: A chairside dental technique. J. Prosthodont..

[20] Gehrke P., Rashidpour M., Sader R., Weigl P. (2024). A systematic review of factors impacting intraoral scanning accuracy in implant dentistry with emphasis on scan bodies. Int. J. Implant Dent..

[21] Karthhik R., Raj B., Karthikeyan B.V. (2022). Role of scan body material and shape on the accuracy of complete arch implant digitalization. J. Oral Res. Rev..

[22] García-Martínez I., Zarauz C., Morejón B., Ferreiroa A., Pradíes G. (2022). Influence of customized over-scan body rings on the intraoral scanning effectiveness of a multiple implant edentulous mandibular model. J. Dent..

[23] Etxaniz O., Amezua X., Jauregi M., Solaberrieta E. (2024). Improving the accuracy of complete arch implant digital scans by using auxiliary clips for intraoral scan bodies: A dental technique. J. Prosthet. Dent..

[24] Pachiou A., Zervou E., Tsirogiannis P., Sykaras N., Tortopidis D., Kourtis S. (2023). Characteristics of intraoral scan bodies and their influence on impression accuracy: A systematic review. J. Esthet. Restor. Dent..

[25] Abdelrehim A., Etajuri E.A., Sulaiman E., Sofian H., Salleh N.M. (2024). Magnitude of misfit threshold in implant-supported restorations: A systematic review. J. Prosthet. Dent..

[26] Demirel M., Donmez M.B., Şahmalı S.M. (2023). Trueness and precision of mandibular complete-arch implant scans when different data acquisition methods are used. J. Dent..

